# Effects of smoking on disease risk among South Korean adults

**DOI:** 10.18332/tid/94472

**Published:** 2018-10-02

**Authors:** Youngmee Kim, Won-Kyung Cho

**Affiliations:** 1 Red Cross College of Nursing, Chung-Ang University, Seoul, South Korea; 2 Department of Pulmonology and Critical Care Medicine, International Health Care Center, Asan Medical Center, University of Ulsan College of Medicine, Seoul, South Korea

**Keywords:** smoking hazard, Korean, COPD

## Abstract

**INTRODUCTION:**

Tobacco smoking is currently considered to be the main preventable cause of death and disability worldwide. We examined the magnitude of cigarette smoking effects on major smoking-related diseases, such as chronic obstructive pulmonary disease (COPD), hypertension (HTN) and cardiovascular disease (CVD), among South Korean adults using nationwide and representative survey data.

**METHODS:**

We used data from the Korea National Health and Nutrition Examination Survey, which was conducted over a 9-year period from 2007 to 2015 by the Korea Centers for Disease Control and Prevention. Multiple logistic regression analyses were performed to assess the associations between smoking status and all outcomes of interest. A total of 24072 participants ≥40 years of age were sampled in the current study.

**RESULTS:**

The study results were as follows: 1) Current and former smoking is associated with lower socioeconomic status; 2) The prevalence of major smoking-related diseases was significantly higher in former and current smokers compared to non-smokers; 3) The odd ratios of developing COPD were 3.49 [95% confidence interval (CI): 2.44–5.00], 2.41 (95% CI: 1.68–3.45) and 3.45 (95% CI: 2.20–5.40) among male current smokers, male ex-smokers and female current smokers, respectively. The odd ratio of developing CVD was 2.01 (95% CI: 1.05–3.86) in male ex-smokers. Otherwise, no significant associations between smoking and other diseases were observed after controlling the sociodemographic and clinical factors; and 4) The risk for COPD tends to be related to the smoking amount and weaning after quitting smoking.

**CONCLUSIONS:**

Our study showed that COPD had the strongest association with current and former smoking in both male and female smokers after controlling for all potential confounding factors among major smoking-related diseases.

## INTRODUCTION

Tobacco smoking is currently considered to be the main preventable cause of death and disability worldwide^1^. Tobacco smoking harms nearly every organ in the body^1,2^; however, millions of people continue to smoke cigarettes. Previous studies have reported that the magnitude of smoking effects on health could vary depending on ethnicity or sex^3-5^. Further, organ-specific smoking effects could also vary depending on ethnicity. In this study, we investigated the prevalence of smoking-related major diseases, associations between smoking and the risks of these diseases, dose-response association between each disease, and smoking and quitting effect of smoking on the risks of these diseases, among South Korean adults using data from the Korea National Health and Nutrition Examination Survey (KNHANES).

## METHODS

### Setting and data sources

This study is a secondary data analysis of KNHANES, which was conducted during a 9-year period from 2007 to 2015 by the Korea Centers for Disease Control and Prevention (KCDC). KNHANES is an ongoing, nationally representative, annual cross-sectional survey that examines the health of the South Korean civilian population^6^. KNHANES is a multistage, stratified and clustered probability sampling survey to reduce the sampling bias, and comprises a health questionnaire, physical and laboratory examinations including pulmonary function tests (PFTs), and chest X-rays. PFTs, which are used for diagnosing chronic obstructive pulmonary disease (COPD), were conducted for individuals aged 40–80 years. Thus, the individuals aged 40–80 years were included in the current study. The participants who had missing data for PFT or smoking history were excluded from the present study. Finally, 24072 participants were sampled in the current study. Data were downloaded from the official website of KNHANES (http://knhanes.cdc.go.kr/) after completing a designated registration process for access^6^.

### Measurements

Sociodemographic characteristics and health behaviours were surveyed using self-reported questionnaires^6^. Regarding the definition of smoking status, we used the guideline of the centers for disease control and prevention (CDC): non-smokers were those who smoked <100 cigarettes in their lifetime; current smokers were those who smoked ≥100 cigarettes in their lifetime and were current smokers at the time of the survey; and ex-smokers were those who smoked >100 cigarettes in their lifetime, but quit smoking at the time of the survey^7,8^. Smoking amount in their lifetime was assessed by multiplying the number of packs of cigarettes smoked per day by the number of years the person smoked (pack-years). Hypertension (HTN) was defined as systolic blood pressure (SBP) of ≥140 mmHg and diastolic blood pressure of ≥90 mmHg, or taking antihypertensive medications. COPD was diagnosed on the basis of an obstructive spirometry pattern secondary to airflow limitation, which is forced expired volume in 1 second (FEV1)/forced vital capacity (FVC) ratio <0.70, according to the Global Initiative for Chronic Obstructive Lung Disease guidelines (GOLD)^9^. Cardiovascular disease (CVD) was defined as a medical history of angina pectoris, acute myocardial infarction, heart failure or stroke based on the physician’s diagnosis. Health-related quality of life was measured using the EuroQoL-5 Dimension (EQ-5D), in which a higher score implies a better quality of life^10^.

### Statistical analyses

Data were analysed using SAS version 9.4 (SAS Institute, Inc., Cary, NC, USA). A p-value of <0.05 was considered to be statistically significant. All data are presented as means ± standard error (SE) for continuous variables or as proportions (±SE) for categorical variables. Analysis of variance and chi-squared test were performed for evaluating the differences in continuous and categorical variables between the groups. The prevalence of each disease was analysed as a proportion (±SE). Multiple logistic regression analyses were performed to explore the associations between smoking and each disease while controlling for all potential variables. The variables with a p-value of <0.05 in the univariate tests in Table 1 (sociodemographic and clinical characteristics of the study participants) were selected for the multivariate test. Therefore, for multiple regression analysis to address an association between each disease and smoking status the following were adjusted for males: age, marital status, currently employed, household income, SBP, BMI, AbA1c, hemoglobin, heavy drink, exercise, perceived health status, perceived psychological stress and EQ-5D; while age, marital status, currently employed, household income, SBP, hemoglobin, heavy drink, perceived health status, perceived psychological stress and EQ-5D were adjusted for females. The results are reported using adjusted odds ratios (ORs) and 95% confidence intervals (CIs). A p-value of <0.05 or 95% CI that did not span 1.0 was considered to be statistically significant.

**Table 1:** Sociodemographic and clinical characteristics of the study participants

	*Male*							*Female*						
	*Non-smoker*		*Current smoker*		*Ex-smoker*		*p*	*Non-smoker*		*Current smoker*		*Ex-smoker*		*p*
**N**	1914		3816		4665			12737		565		375		
% of N	18.4%		36.7%		44.9%			93.1%		4.1%		2.8%		
Age	54.8	±0.29	52.0	±0.18	57.2	±0.20	<.0001	56.1	±0.14	53.9	±0.57	57.2	±0.91	0.0003
**Age grouping**							<.0001							<.0001
40–49	38.6%	(1.41)	47.5%	(0.99)	28.9%	(0.88)		33.9%	(0.59)	44.4%	(2.57)	37.4%	(3.12)	
50–59	30.4%	(1.30)	32.8%	(0.91)	31.1%	(0.85)		29.6%	(0.49)	28.9%	(2.26)	20.4%	(2.35)	
60–69	18.0%	(0.94)	13.1%	(0.58)	23.5%	(0.71)		20.8%	(0.44)	12.6%	(1.41)	16.9%	(2.01)	
70–79	13.0%	(0.81)	6.6%	(0.42)	16.4%	(0.64)		15.8%	(0.45)	14.1%	(1.81)	25.3%	(3.04)	
**Marital status**							<.0001							<.0001
Married	93.0%	(0.72)	86.1%	(0.74)	92.6%	(0.49)		80.7%	(0.44)	58.1%	(2.43)	56.7%	(3.04)	
Never-married	3.1%	(0.53)	5.3%	(0.49)	1.9%	(0.26)		1.0%	(0.11)	3.7%	(0.82)	4.0%	(1.22)	
Living without spouse/partner	3.9%	(0.54)	8.6%	(0.58)	5.5%	(0.42)		18.3%	(0.43)	38.3%	(2.35)	39.2%	(3.04)	
Currently employed	82.7%	(1.00)	84.2%	(0.69)	74.7%	(0.80)	<.0001	50.1%	(0.62)	49.8%	(2.59)	40.8%	(3.13)	0.0176
**Household income (Quartiles)**							<.0001							<.0001
Q1 (Lowest)	24.4%	(1.21)	28.1%	(0.94)	20.6%	(0.76)		23.6%	(0.51)	43.4%	(2.58)	33.1%	(2.84)	
Q2	22.0%	(1.18)	27.1%	(0.89)	25.4%	(0.79)		25.2%	(0.48)	26.6%	(2.14)	29.4%	(2.86)	
Q3	24.1%	(1.14)	24.2%	(0.84)	27.0%	(0.78)		25.6%	(0.47)	15.7%	(1.78)	22.3%	(2.45)	
Q4 (Highest)	29.5%	(1.31)	20.7%	(0.81)	27.0%	(0.87)		25.5%	(0.57)	14.3%	(1.81)	15.1%	(2.18)	
**Cigarette smoking amount**							0.0085							<.0001
<10 cigarettes/day			12.4%	(0.61)	12.6%	(0.56)				42.4%	(2.65)	57.2%	(3.05)	
10–19 cigarettes/day			33.2%	(0.89)	29.6%	(0.83)				42.0%	(2.61)	26.3%	(2.62)	
≥20 cigarettes/day			54.3%	(0.95)	57.8%	(0.88)				15.6%	(1.74)	16.5%	(2.03)	
Smoking duration (years)			32.0	±0.21	21.5	±0.21	<.0001			23.0	±0.67	12.0	±0.74	<.0001
Lifetime smoking amount (pack-years)			28.0	±0.36	21.9	±0.33	<.0001			11.8	±0.61	6.7	±0.57	<.0001
**Duration of smoking cessation**					171.6	±2.27						161.1	±7.99	
< 15 years					58.2%	(0.86)						60.1%	(3.05)	
≥15 years					41.8%	(0.86)						39.9%	(3.05)	
SBP (mmHg)	121.3	±0.42	121.0	±0.32	123.4	±0.29	<.0001	120.3	±0.21	117.7	±0.97	120.5	±1.05	0.0345
DBP (mmHg)	79.3	±0.27	79.5	±0.22	79.3	±0.20	0.7606	75.4	±0.12	74.4	±0.55	75.2	±0.55	0.2358
BMI (kg/m^2^)	24.4	±0.08	24.2	±0.06	24.6	±0.05	<.0001	24.2	±0.04	24.0	±0.20	24.2	±0.24	0.8275
Waist circumference (cm)	85.2	±0.23	85.5	±0.16	86.6	±0.14	<.0001	80.9	±0.12	81.4	±0.56	81.8	±0.62	0.2576
Fasting blood sugar (mg/dL)	104.4	±0.86	103.6	±0.49	104.7	±0.46	0.2515	99.1	±0.25	98.6	±1.15	99.8	±1.45	0.8036
HbA1C	6.0%	±0.04	6.1%	±0.03	6.0%	±0.02	0.0463	5.9%	±0.01	5.9%	±0.06	5.9%	±0.06	0.6179
Hemoglobin (g/dL)	15.1	±0.03	15.4	±0.02	15.0	±0.02	<.0001	13.1	±0.01	13.4	±0.06	13.1	±0.08	0.0001
Alcohol drinking	16.1%	(1.19)	34.1%	(0.97)	22.4%	(0.82)	<.0001	4.0%	(0.28)	22.0%	(2.45)	12.1%	(2.58)	<.0001
Moderate physical exerciser	23.7%	(1.40)	21.9%	(0.96)	26.2%	(0.92)	0.0034	20.3%	(0.56)	16.7%	(2.25)	16.9%	(2.62)	0.1695
**Perceived health status**							<.0001							<.0001
Very good/Good	44.5%	(1.35)	32.6%	(0.90)	37.7%	(0.85)		29.3%	(0.51)	22.8%	(2.14)	24.5%	(2.73)	
Fair	43.2%	(1.33)	50.3%	(0.96)	46.4%	(0.92)		46.9%	(0.55)	41.9%	(2.64)	40.6%	(3.02)	
Poor/Very poor	12.3%	(0.89)	17.0%	(0.74)	15.9%	(0.65)		23.8%	(0.47)	35.3%	(2.49)	35.0%	(3.21)	
EQ-5D	0.96	±0.002	0.96	±0.002	0.96	±0.002	0.0093	0.92	±0.002	0.89	±0.008	0.87	±0.013	<.0001
Perceived psychological stress	18.1%	(1.05)	27.1%	(0.85)	17.8%	(0.65)	<.0001	24.8%	(0.46)	41.6%	(2.56)	34.1%	(2.85)	<.0001

Values are sample n, weighted mean ± SE or sample n, weighted percentage (SE) unless otherwise indicated; † p-value by ANOVA or chi-squared test as appropriate; Household income quartiles (Q) are adjusted for age and gender. A total of 24072 Koreans were sampled from 2007 to 2015.

## RESULTS

### Characteristics of study participants

Table 1 presents the sociodemographic and clinical characteristics of the study participants. Among the male participants, current smokers, ex-smokers and non-smokers were 36.7%, 44.9% and 18.4%, respectively. The mean age of ex-smokers was 57.2 years, which was higher than that of current smokers (52.0 years) or non-smokers (54.8 years) (p<0.001). Overall, compared to non-smokers, both male current and former smokers tended to be unmarried, had poorer incomes, lower quality of life, poorer self-perceived health status, higher perceived psychological stress and consumed more alcohol. Among the female participants, current smokers, ex-smokers and non-smokers were 4.1%, 2.8% and 93.1%, respectively. In females, the mean age of ex-smokers (57.2 years) was higher than that of current smokers (53.9 years) or non-smokers (56.1 years) (p<0.001). Overall, compared to non-smokers, both female current and former smokers had poorer incomes, consumed more alcohol and had higher perceived psychological stress.

### Prevalence of smoking-related diseases

Table 2 presents the prevalence of smoking-related major diseases among the study participants. Among males, COPD was more prevalent among ex-smokers (22.4%) and current smokers (20.7%) than among non-smokers (13.2%) (p<0.001). The prevalence of HTN (43.2%) and CVD (6.4%) was the highest among ex-smokers and the lowest among current smokers. Among females, COPD was more prevalent among ex-smokers (14.1%) and current smokers (12.7%) than among non-smokers (6.1%) (p<0.001). There was no difference in the prevalence of HTN and CVD according to smoking status among females.

**Table 2:** Prevalence of smoking-related major diseases

	*Male*							*Female*						
	*Non-smoker*		*Current smoker*		*Ex-smoker*		*p*	*Non-smoker*		*Current smoker*		*Ex-smoker*		*p*
COPD (%)	13.2%	(0.88)	20.7%	(0.75)	22.4%	(0.72)	<.001	6.1%	(0.27)	12.7%	(1.63)	14.1%	(2.40)	<.001
HTN (%)	37.3%	(1.29)	34.8%	(0.94)	43.2%	(0.87)	<.001	33.9%	(0.56)	30.0%	(2.38)	35.1%	(3.21)	0.299
CVD (%)	3.4%	(0.45)	2.9%	(0.29)	6.4%	(0.43)	<.001	3.6%	(0.19)	4.0%	(0.90)	5.3%	(1.25)	0.301

Values are sample n, weighted percentage (SE); † p-value by chi-squared test. A total of 24072 Koreans were sampled from 2007 to 2015.

### Association between smoking and major smoking-related diseases

**Figure 1:**
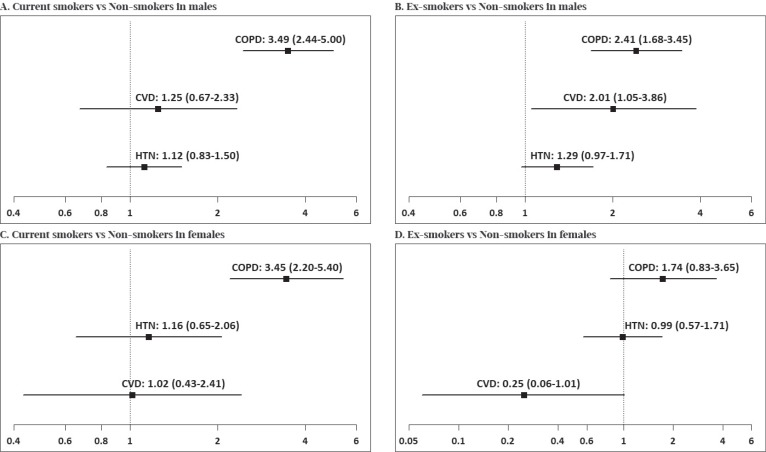
Associations between smoking and the risk of the major smoking-related diseases based on multivariate-adjusted odd ratios and their 95% confidence intervals. Male: Adjusted for age, marital status, currently employed, household income, SBP, BMI, AbA1c, Hemoglobin, heavy drink, exercise, perceived health status, perceived psychological stress and EQ-5D.Female: Adjusted for age, marital status, currently employed, household income, SBP, hemoglobin, heavy drink, perceived health status, perceived psychological stress and EQ-5D. A total of 24072 Koreans were sampled from 2007 to 2015

To address the associations between smoking and the risks for major smoking-related diseases, ORs were calculated after controlling for the sociodemographic and clinical factors. Fig.1A shows that OR for developing COPD among male current smokers was 3.49 (95% CI: 2.44–5.00) times higher than that among male non-smokers. Otherwise, no significant association between other diseases and smoking was observed among male current smokers. Fig.1B shows that OR for developing COPD among male ex-smokers was 2.41 (95% CI: 1.68–3.45) times higher than that among male non-smokers. In addition, OR for developing CVD among male ex-smokers was 2.01 (95% CI: 1.05–3.86) times higher than that among male non-smokers. Furthermore, a tendency for an association between former smokers and HTN was observed among male ex-smokers. Fig.1C shows that OR for developing COPD was 3.45 (95% CI: 2.20–5.40) times higher among female current smokers than that among female non-smokers. No significant association was observed between major smoking-related diseases and previous smoking among females, but a tendency for an association between former smokers and CVD was observed among female ex-smokers (Fig.1D). Overall, COPD had the strongest association with smoking among Korean smokers based on adjusted ORs. There was a significant association between CVD and previous smoking among males only. Otherwise, the associations between smoking and other diseases were not observed.

### Dose-response relationship between smoking amount and disease risks

To address the dose-response association between cigarette consumption and the risk for a disease, adjusted ORs were calculated based on smoking amount (pack-years), with the 0–10 pack-year smoking as the reference value. As shown in Table 3, the overall risk for COPD was not affected by the smoking amount among males. However, ORs for developing COPD were 1.96 (95% CI: 1.25–3.07) and 1.79 (95% CI: 1.22–2.63) in the combined group of current and former smokers with 20–30 or ≥30 pack-year smoking history, respectively, suggesting a potential dose response. There was a significant dose-response association between cigarette consumption and the risk for HTN (p=0.0016). In addition, OR for CVD was 1.98 (95% CI: 1.02–3.84) in the combined group of smokers with ≥30 pack-year smoking history, implicating a possible dose response. Among females, the disease risks were not affected by the smoking amount (Table 3). However, OR for COPD among the combined group of smokers with ≥20 pack-year smoking history was significantly higher, which was 3.92 (95% CI: 1.30–11.81), suggesting a potential dose response. In summary, the risk for HTN showed a dose-response association with smoking among males. Furthermore, the risk for COPD among both males and females and the risk for CVD among males might have a dose-response association with cigarette consumption. Next, we calculated ORs based on the duration of smoking cessation (<15 years vs ≥15 years) among ex-smokers to observe the quitting effects of smoking on the risk for each disease. Among males, quitting smoking for >15 years was significantly associated with lower OR for COPD (OR=0.65; 95% CI: 0.47–0.91). Otherwise, no quitting effect was observed (Table 4).

**Table 3:** Effect of smoking amount on the risks of diseases

	*Male*						*Female*					
	*Current smoker*		*Ex-smoker*		*Both current and ex-smoker*		*Current smoker*		*Ex-smoker*		*Both current and ex-smoker*	
**COPD**	**Adjusted OR^†^**	**95% CI**	**Adjusted OR^†^**	**95% CI**	**Adjusted OR^†^**	**95% CI**	Adjusted OR*	**95% CI**	**Adjusted OR^†^**	**95% CI**	**Adjusted OR^†^**	**95% CI**
0–10	1		1		1		1		1		1	
10–20	0.63	(0.31–1.29)	1.51	(0.90–2.54)	1.21	(0.79–1.85)	0.74	(0.23–2.39)	1.35	(0.27–6.65)	0.85	(0.31–2.29)
20–30	1.27	(0.63–2.54)	2.21	(1.27–3.86)	1.96	(1.25–3.07)	4.55	(1.37–15.08)	2.58	(0.29–23.28)	3.92	(1.30–11.81)
≥30	1.19	(0.64–2.21)	1.99	(1.23–3.21)	1.79	(1.22–2.63)						
**HTN**												
0–10	1		1		1		1		1		1	
10–20	2.92	(1.35–6.30)	0.89	(0.59–1.36)			1.39	(0.48–4.02)	1.83	(0.35–9.47)	1.53	(0.60–3.89)
20–30	2.92	(1.49–5.71)	0.63	(0.41–0.96)			0.37	(0.10–1.33)	1.05	(0.16–7.07)	0.52	(0.17–1.62)
≥30	2.33	(1.19–4.55)	0.73	(0.47–1.11)								
**CVD**												
0–10	1		1		1		1		1		1	
10–20	1.30	(0.26–6.46)	1.65	(0.75–3.67)	1.65	(0.79–3.41)	Nonestimable		Nonestimable		0.75	(0.01–45.56)
20–30	1.02	(0.20–5.30)	2.44	(0.97–6.12)	2.04	(0.92–4.56)	Nonestimable		Nonestimable		1.62	(0.11–22.96)
≥30	1.40	(0.34–5.71)	2.05	(0.97–4.37)	1.98	(1.02–3.84)						

† Adjusted for age, marital status, currently employed, household Income, SBP, BMI, HbA1c, Hemoglobin, heavy drink, exercise, perceived health status, perceived psychological stress and EQ-5D in males; p=0.2802, 0.0016 and 0.8132 for interaction between smoking status and pack years in COPD, HTN and CVD, respectively, among males.

* Adjusted for age, marital status currently employed, household Income, SBP, Hemoglobin, heavy drink, perceived health status, perceived psychological stress and EQ-5D in females; p=0.6622 and 0.6422 for interaction between smoking status and pack years in COPD and HTN, respectively, among females. A total of 24072 Koreans were sampled from 2007 to 2015.

**Table 4:** Effect of smoking cessation on the risk for diseases

	*Male*			*Female*		
	*<15 years*	*≥ 15 years*		*<15 years*	*≥ 15 years*	
*Duration of cessation*		*Adjusted OR^†^*	*95% CI*		*Adjusted OR^‡^*	*95% CI*
COPD	1	0.65	(0.47-0.9)	1	1.49	(0.41-5.3)
Hypertension	1	1.19	(0.87-1.64)	1	2.09	(0.66-6.62)
CVD	1	0.68	(0.39-1.17)	1	Non-estimable	

† Adjusted for age, marital status, currently employed, household Income, SBP, BMI, HbA1c, Hemoglobin, alcohol drink, exercise, perceived health status, perceived psychological stress, and EQ-5D;

‡ Adjusted for age, marital status, currently employed, household Income, SBP, Hemoglobin, alcohol drink, perceived health status, perceived psychological stress and EQ-5D. A total of 24072 Koreans were sampled from 2007 to 2015.

## DISCUSSION

The current study aimed to investigate the prevalence of smoking-related major diseases, risks for developing these diseases and dose-response association between smoking and the risks for these diseases among South Korean adults using the nationally representative samples from KNHANES. The findings of our study can be summarized as follows: 1) the smoking rate of Korean males was similar to the global rate, but that of Korean females was a lot lower than the global rate; 2) the prevalence of the major smoking-related diseases was higher in former and current smokers compared to non-smokers; 3) COPD was the only disease with a significant association with current and former smoking in both male and female smokers after controlling for all potential confounding factors; and 4) males appeared to be more susceptible to smoking hazards than females.

First, regarding the characteristics of the study participants, the current smoking rate of males was 36.7%, which is comparable to the global rate, but that of females was 4.1%, which is lower than the global rate^11^. As previously reported, socioeconomic status levels among Korean smokers were also lower than those among Korean non-smokers^11^. Thus, our study participants appeared to represent a typical group of smokers. However, there are a few interesting points about our study population. Smoking tends to be much more strongly associated with low income among women than men in our study participants, as opposed to the previous study that addressed low socioeconomic status on smoking^12^.

Second, we examined the prevalence of major smoking-related diseases, COPD, HTN and CVD. We observed that the prevalence of these diseases was significantly higher among current smokers and/or ex-smokers than among non-smokers. Thus, not surprisingly, these findings implicate the harmful effects of smoking on health in our study participants. We further observed that the prevalence of most diseases among ex-smokers was higher than that among current smokers, suggesting that the development of a disease might have been a motivation to quit. In addition, the prevalence of smoking-related diseases was higher among males overall. In general, smoking is considered to have similar adverse health effects for women and men. However, few studies suggest that women are more susceptible to developing smoking-related diseases^13-15^. As an example, several studies have indicated that for a given number of cigarettes smoked, females might be at greater risk in developing lung cancer compared to men^16^. Based on our study findings, males appear to be more susceptible to developing smoking-related disease among Korean smokers. However, the results could be simply because of a smaller amount of lifetime cigarette consumption by female smokers.

Third, we addressed an association between smoking and the risks for developing diseases by calculating multivariate adjusted ORs. Although the effects of smoking on the risks for developing CVD has been suggested, surprisingly, other than COPD, a significant smoking effect on the risk for other diseases was not observed among Korean smokers. Cigarette smoking has been shown to harm nearly every organ in the human body^1^. For instance, the US Department of Health and Human Services estimate that smoking increases the risk for coronary heart disease by 2–4 times, stroke by 2–4 times, men developing lung cancer by 25 times and women developing lung cancer by 25.7 times^2^. Therefore, our study findings are rather surprising. Another method to address the smoking effect on health was to examine the dose-response effect of smoking on health. It is possible that the slightly milder smoking effect on health among Koreans was because of a small smoking amount among the study participants. When we addressed this, we observed a dose-response association between cigarette consumption and the risk for HTN among males only. However, because there was no significant association between smoking and the risk for HTN among males in the current study, the interpretation of data remains unclear. We also observed a probable dose-response association between cigarette consumption and the risk for COPD among both males and females and the risk for CVD among males. The dose-response effect of smoking on COPD has been also previously implicated. A previous study suggested that the single best determinant for predicting the development of COPD is >40 pack-year smoking history^17^. Of note, in this study, we chose the smokers with 0–10 pack-year smoking history, not non-smokers, as the reference value to address the dose-response relationship. This is to avoid any potential unnecessary confounding factors to address the role of smoking amount in the development of COPD, since COPD can develop even among non-smokers. A previous study reported that smokers who quit for ≥15 years no longer experienced an increased risk for most smoking-related diseases^18-20^. We also observed that the risk for COPD appears to be decreased after quitting because smoking cessation for >15 years was significantly associated with low ORs among male smokers. Besides COPD, there was no difference in terms of smoking cessation effect in the risk for other diseases. Overall, there appears to be a rather solid, although incomplete, association between COPD and smoking in terms of the risk for disease, dose-response association and quitting effect in our study participants.

Only 15–20% of smokers are known to develop clinically significant COPD^21^. In the present study, the prevalence of COPD was 20.7% and 22.4% among male current smokers and ex-smokers, respectively, and 12.7% and 14.1% among female current smokers and ex-smokers, respectively. Thus, the prevalence of COPD appears to be comparable to that of the reported global COPD among male Korean smokers, but is lower among female Korean smokers. There are a few points worth mentioning regarding the diagnosis of COPD in our study. First, COPD was diagnosed based on an obstructive spirometry pattern secondary to airflow limitation according to the GOLD guidelines, which means people with subclinical diseases without symptoms might have been included in our study. Second, only participants aged ≥40 years were included in this study; thus, COPD patients younger than 40 years could have been missed, although it is less likely as ageing plays a key role in the pathogenesis of COPD^22^. Third, to diagnose COPD definitively, post-bronchodilator spirometry data are needed to exclude patients with reversible airflow limitation, such as asthma. However, the KNHANES data do not include post-bronchodilator spirometry results. Therefore, we believe that some patients with asthma might have been included in this study^9^. We acknowledge that it is possible that all these factors could have affected the prevalence of COPD in our study. In the past, a few researchers reported that the risk for COPD varies according to ethnicity, and a higher risk for COPD has been reported in Whites compared to all non-White ethnic groups^4^, although ethnicity difference has been considered to be present owing to confounding factors, such as risk factor exposure and access to health care, among others^3^. Previous studies have also proposed possible mechanisms to explain the individual difference in terms of smoking effect on health, e.g. variation in the metabolism of nicotine, difference in the depth of inhaled nicotine per cigarette smoked or dietary intake of fruit and vegetables^23-26^. For now, we are not certain why the harmful effect of smoking on health is not as serious as expected among Korean smokers.

The main strength of this study is that it was done with a large number of individuals using nationwide and representative survey data. However, the present study has several limitations. Our study is based on self-reported data. Thus, there may have been inconsistencies in the self-reported levels of smoking. Moreover, we lack detailed smoking history, such as age at initiation of smoking or smoking habits of participants, which could have significantly affected our results. Finally, the cross-sectional nature of our analyses precludes examination of the associations between smoking and chronic disease outcomes, which can only be examined using longitudinal data. In other words, we could only draw conclusions about associations and not about causation, as with any observational study.

## CONCLUSIONS

In the present study, we examined the associations between smoking and the risks for major smoking-related diseases in a systemic manner using the national survey data among Koreans ≥40 years of age and found that COPD had the strongest association with current and former smoking in both male and female smokers, after controlling for all potential confounding factors among major smoking-related diseases. There was also a significant association between CVD and previous smoking among males only. Otherwise, associations between smoking and other diseases were not observed.

## CONFLICTS OF INTEREST

Authors have completed and submitted the ICMJE Form for Disclosure of Potential Conflicts of Interest and none was reported.
